# Idiopathic True Radial Artery Aneurysm: A Rare Case Report

**DOI:** 10.34172/aim.2023.34

**Published:** 2023-04-01

**Authors:** Zoran Damnjanovic, Nemanja Stepanovic, Nikola Zivkovic

**Affiliations:** ^1^Department for Surgery, Anesthesiology and Reanimatology, Faculty of Medicine, University of Nis, Nis, Serbia; ^2^Vascular Surgery Clinic, Clinical Center of Nis, Nis, Serbia; ^3^Department of Pathology, Faculty of Medicine, University of Nis, Nis, Serbia

**Keywords:** Aneurysm, Radial artery, Treatment

## Abstract

Idiopathic true aneurysm of the distal radial artery is a rare disease with only few reported cases. Most patients were treated surgically with proximal and distal arterial ligatures, while there are reports of only 7 cases where revascularization procedures were performed. We present a case of a 66-year-old man with a pulsatile mass in the right forearm at the location of the radial artery. Six months preceding the presentation, the patient had first noticed a pulsatile tumefaction which gradually increased in size, with a sudden increase during the last month. The patient worked as a waiter and was a non-smoker with no significant comorbidities. There was no history of trauma, recent infection, hospitalization, recurrent injury, or peripheral venous cannulation. After CDT diagnosis, we performed resection of aneurysm and reconstruction with cephalic autovenous graft. One month afterwards, at the follow-up visit, the patient denied having symptoms of hand ischemia and duplex ultrasound examination showed adequate patency of the radial artery. This paper presents a rare case of a true idiopathic radial artery aneurysm that was treated surgically by complete resection and interposition with a reverse cephalic vein autovenous graft. Detailed anamnesis and clinical examination are necessary for the appropriate surgical treatment of the disease.

## Introduction

 Idiopathic true aneurysms of the distal radial artery represent a rare disease with only 12 reported caseswhere most patients were treated surgically with proximal and distal arterial ligatures,^1-4^ while there are reports of only 7 cases where revascularization procedures were performed.^1-7^ This paper presents a case of true idiopathic aneurysm of the radial artery with description of the pathological and angiographic findings, as well as the method of surgical treatment of the disease.

## Case Report

 The presentation involves a 66-year-old man with a pulsatile mass in the right forearm at the location of the anatomical position of the radial artery. Six months preceding the presentation, the patient had first noticed a pulsatile tumefaction which gradually increased in size, with a sudden increase during the last month. The patient worked as a waiter and was a non-smoker with no significant comorbidities. There was no history of trauma, recent infection, hospitalization, recurrent injury, or peripheral venous cannulation. Clinical examination revealed a pulsating mass in the right forearm, which filled rapidly after releasing pressure, without auscultatory systolic murmur and palpable thrill. The patient reported no significant subjective complaints. The laboratory parameters were within reference values. Allen’s test was abnormal.

 Doppler sonography revealed a fusiform radial artery aneurysm; then CT angiography of the right arm was performed, which confirmed the presence of a distal radial artery aneurysm measuring 11 × 12.5 at 12 cm from the wrist.

**Figure 1:**
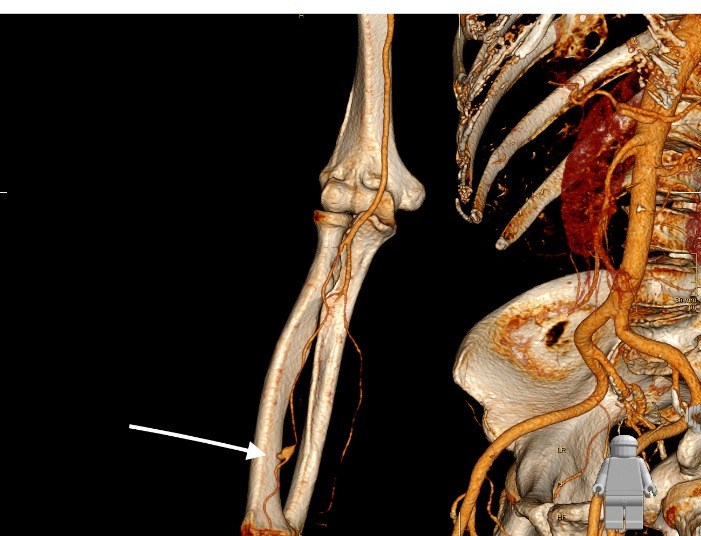
3D Reconstruction of Multi-detector Computed Tomography (MDCT) Showing True Aneurysm of Radial Artery. Arrow shows aneurysm.

**Figure 2:**
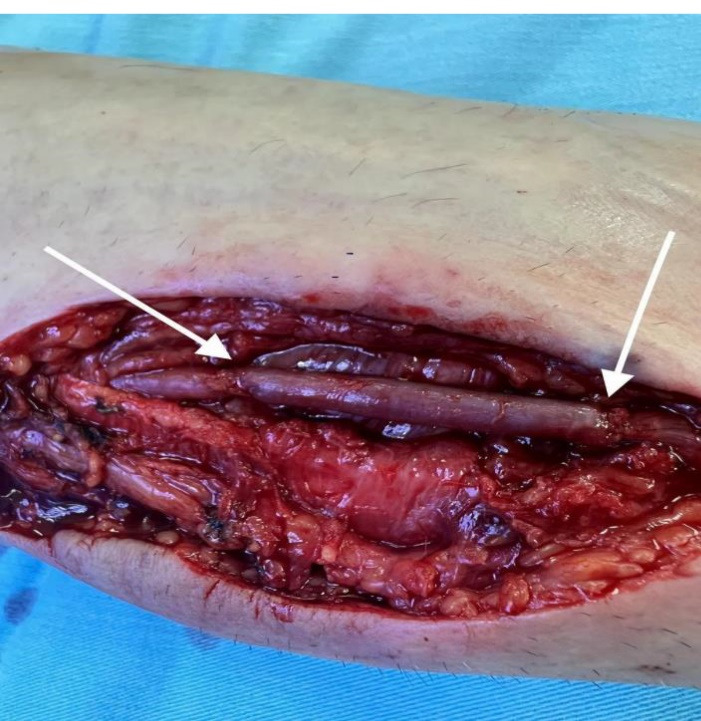
Intraoperative Image Showing Implanted Reversed Saphenous Graft with Transanal Tube Anastomoses. Arrows show anastomoses of vein graft.

**Figure 3:**
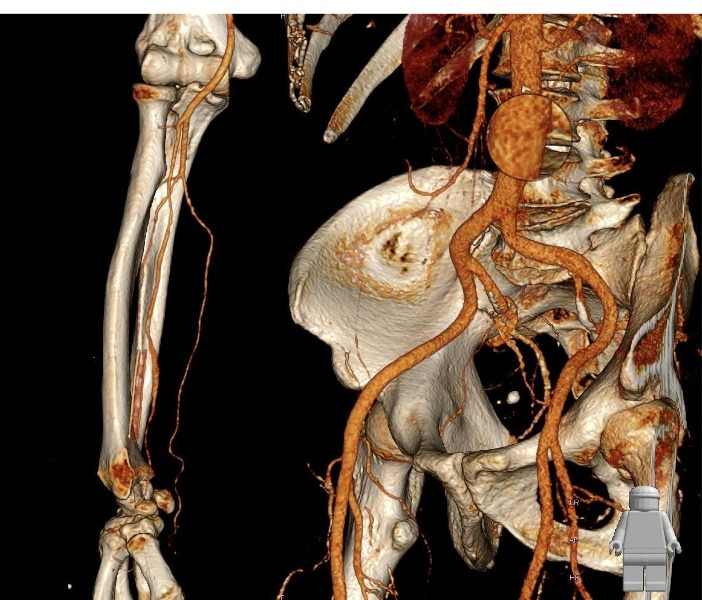
Control 3D Reconstructed Multi-detector Computed Tomography (MDCT) Showing Patent Vein Graft.

**Figure 4:**
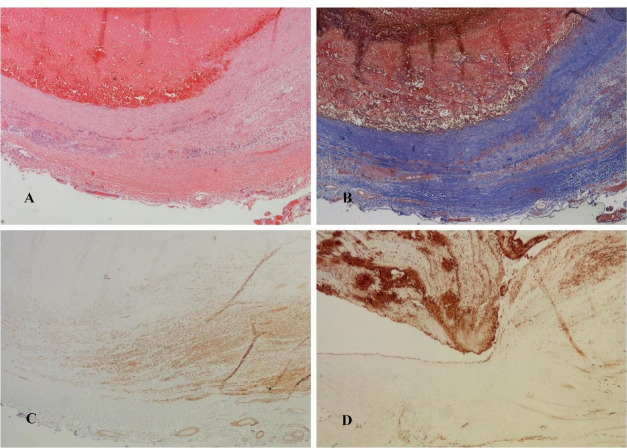
Histology of Aneurysm. (A) Hematoxylin-eosin, magnification × 40; (B) Masson’ Trichrome, magnification × 40; (C) Actin, magnification × 40; (D) CD31, magnification × 100.

 Considering the sudden increase in the diameter of the aneurysm, the probability of thromboembolic complications and rupture, as well as hand ischemia that would occur in case of complete occlusion of the aneurysm (which would be expected due to the abnormal Allen’s test), the patient underwent a revascularization procedure. Under local anesthesia, the anatomical position of the radial artery aneurysm was approached through a longitudinal incision. Complete resection of the aneurysm was performed. Due to the length of the aneurysm, we decided against performing a primary end-to-end anastomosis of the radial artery after aneurysm resection and instead opted for reconstruction with reverse autovenous cephalic vein graft interposition (Figures 1 and 2). The operation was completed without complications with a satisfactory revascularization effect, and the aneurysm tissue was sent for pathohistological examination. Control MSCT angiography showed patency of the existing graft and adequate flow in the radial artery (Figure 3). Both micromorphological analysis on histological sections processed by the standard H&E (hematoxylin-eosin) method and histochemical analysis by the Masson’s trichrome method, showed all three layers in the structure of the muscle-type artery, which revealed that it was undoubtedly a true aneurysm of the radial artery (Figure 4). The endothelium was preserved and confirmed by CD31 and CD34 immunohistochemical staining. The central layer, confirmed by the expression of antibodies to Actin and Desmin, showed thinning which led to the consequent formation of the aneurysm. Adventitia was preserved on the periphery.

 One month afterwards, at the follow-up visit, the patient denied having symptoms of hand ischemia and duplex ultrasound examination showed adequate patency of the radial artery.

## Discussion

 The etiopathogenetic mechanism of true idiopathic aneurysms of the radial artery still remains insufficiently understood. However, this rare disease has the same risk of complications as other aneurysms. Distal ischemia, as a potential complication, may be the result of thrombus embolization within the aneurysm or thrombus propagation to the distal artery.^1-7^

 According to the analysis of the previous literature data on the reconstructive surgical treatment of true idiopathic radial artery aneurysm,^1-7^ resection with revascularization via end-to-end anastomosis had been performed in four cases,^1,2,4,7^ while reconstruction by resection and interposition of a vein graft was reported in three cases^3,5,6^ with autovenous cephalic vein graft used twice,^3,6^ and saphenous graft used only once.^5^ Some authors decided to reconstruct the artery even with a normal Allen’s test.^3^

 Determining an adequate surgical treatment in the treatment of radial artery aneurysm comes down to an individual approach to the patient, whereby the surgeon’s decision is guided by the analysis of whether the radial or ulnar artery carries the greater part of the hand’s blood supply. In addition to detailed analysis of angiographic findings to determine the surgical treatment strategy in patients with true idiopathic aneurysm of the radial artery, Allen’s test is very important due to the fact that it determines the potency of the superficial arterial arch and the collateral circle between the ulnar and radial arteries. If the patency of the collateral network is not adequate, it is necessary to perform resection and revascularization of the existing radial artery aneurysm, and if it is patent, resection of the aneurysm and ligature of the radial artery is possible.

 In conclusion, this paper presents a rare case of a true idiopathic radial artery aneurysm that was treated surgically by complete resection and interposition with a reverse cephalic vein autovenous graft. Detailed anamnesis and clinical examination are necessary for the appropriate surgical treatment of the disease.

## References

[1] Taengsakul N (2022). Idiopathic true aneurysm of distal radial artery aneurysm in Chulabhorn hospital: a case report. Ann Vasc Surg Brief Rep Innov.

[2] Erdogan SB, Akansel S, Selcuk NT, Aka SA (2018). Reconstructive surgery of true aneurysm of the radial artery: a case report. North Clin Istanb.

[3] Madeline Chee YM, Lew PS, Darryl Lim MJ (2020). True idiopathic radial artery aneurysm: a case report and review of current literature. EJVES Vasc Forum.

[4] Al-Zoubi NA (2018). Idiopathic true aneurysm of distal radial artery: case report. Vasc Health Risk Manag.

[5] Ghaffarian AA, Brooke BS, Rawles J, Sarfati M (2018). Repair of a symptomatic true radial artery aneurysm at the anatomic snuff box with interposition great saphenous vein graft. J Vasc Surg Cases Innov Tech.

[6] Ayers JD, Halbach J, Brown D (2015). True radial artery aneurysm: diagnosis and treatment. J Vasc Surg.

[7] Malt S (1978). An arteriosclerotic aneurysm of the hand. Arch Surg.

